# Allelopathic Effects of Litter *Axonopus compressus* against Two Weedy Species and Its Persistence in Soil

**DOI:** 10.1155/2013/695404

**Published:** 2013-10-23

**Authors:** B. Samedani, A. S. Juraimi, M. Y. Rafii, A. R. Anuar, S. A. Sheikh Awadz, M. P. Anwar

**Affiliations:** ^1^Department of Crop Science, Faculty of Agriculture, Universiti Putra Malaysia, 43400 UPM, Serdang, Selangor, Malaysia; ^2^Institute of Tropical Agriculture, Universiti Putra Malaysia, 43400 UPM, Serdang, Selangor, Malaysia; ^3^Department of Land Management, Faculty of Agriculture, Universiti Putra Malaysia, 43400 UPM, Serdang, Selangor, Malaysia

## Abstract

This study investigated the allelopathic effect of *Axonopus compressus *litter on *Asystasia gangetica *and *Pennisetum polystachion*. In experiment 1 the bioassays with 0, 10, 30, and 50 g L^−1^ of aqueous *A. compressus *litter leachate were conducted. Experiment 2 was carried out by incorporating 0, 10, 20, 30, 40, and 50 g L^−1^ of *A. compressus *litter leachate into soil. In experiment 3, the fate of *A. compressus *litter leachate phenolics in the soil was investigated. *A. compressus *leachates did not affect the germination percentage of *A. gangetica *and *P. polystachion*, but delayed germination of *A. gangetica *seeds and decreased seed germination time of *P. polystachion*. *A. compressus* litter leachates affected weeds hypocotyl length. Hypocotyl length reductions of 18 and 31% were observed at the highest concentration (50 g L^−1^) compared to the control in *A. gangetica *and *P. polystachion*, respectively. When concentration of * A. compressus *litter leachate-amended soil increased *A. gangetica *and *P. polystachion *seedling shoot length, root length, seedling weight and chlorophyll concentration were not affected. The 5-week decomposition study of *A. compressus *showed that the phenolic compounds in *A. compressus *litter abruptly decreased about 52% after two weeks and remained steady until the end of the incubation.

## 1. Introduction

Currently oil palm occupies the largest acreage of farmed land in both Malaysia and Indonesia having overtaken rubber and coconuts, respectively. Oil palm covered more than 12 million ha in the world in 2007, a 50% increase over the past 10 years, with Malaysia having 41% and Indonesia 44% of the total [1]. Palm oil is the largest internationally traded vegetable oil in the main markets in China, European Union, Pakistan, India, Japan, and Bangladesh [2].

Weeds are the major components in the oil palm production system. Weeds grow luxuriantly under the tropical conditions of high rainfall and relative humidity and temperature [3]. In Malaysia, 60 to 70 weed species are found to be growing under young oil palms, of which 20 to 30 species remain under older trees [4, 5]. These weeds are able to strongly compete with oil palm for soil nutrients, soil water, light, and space [6]. The use of herbicides to control weeds is a common practice and extensive in oil palm plantations in Malaysia [7, 8].

Conventional legume cover crops (LCC) such as *Centrosema pubescens, Pueraria phaseoloides, *and *Calopogonium caeruleum* have long been widely cultivated under rubber and oil palm, while *Mucuna bracteata* was introduced into oil palm plantations only in 1991 [9]. The conventional legume cover crops become shaded out, and soft grasses such as *Axonopus compressus*, *Cytococcum *sp., and *Paspalum conjugatum *and light ferns cover the field. Finally, noxious weeds like *Asystasia* and *Mikania *can dominate in these areas because of their high tolerance to low soil fertility and shade from the palm canopy [10]. *Mucuna bracteata* has a climbing habit and requires regular pruning around the tree base to prevent it from smothering the tree crop. Hence, maintenance of *M. bracteata* can be labour-intensive. 

In Malaysia, *A. compressus* is one of the soft-grass species that is widely used as ground cover to protect soil erosion, as turf grass for landscaping and for sports fields, as well as to conserve soil moisture [11]. *A. compressus* could compete against *Asystasia gangetica* and was less susceptible to *Pennisetum polystachion* interference than the legumes cover crops [12]. Samedani et al. (data not published) also showed that *A. compressus* increased oil palm yield 11–35% compared to legume cover crops. Rika et al. [13] found that the coconut yield was highest when *A. compressus* was used as ground cover under coconut plantations compared with other grass species used as ground cover. This grass has a high potential for use as a cover crop to suppress weeds in plantations, especially areas that are dominated by broadleaf weeds and where establishing legume cover crops is not feasible. 

*A. compressus* is known to produce allelochemicals affecting the growth of other plants [14, 15]. Weed and crop interference has two components, competition and allelopathy. Both components cannot be differentiated in the field [16]; however, it is possible to show allelopathic effects using *in vitro* studies. If allelopathy is to be a profitable weed-control measure, then its research requires greater accuracy. From an allelopathic perspective, phytotoxic compounds are not considered suitable if they are not released into their environment, and the fate of the allelochemicals in the soil should be considered [17]. Therefore, we hypothesized that *A. compressus *might control weeds with allelopathic properties, and this research was designed to evaluate the potential phytotoxic effects of *A. compressus *on weed species commonly associated with it.

## 2. Materials and Methods

### 2.1. Plant Material

Litter was collected from the top layer of the litter beds of growing populations of *A. compressus* from oil palm fields. In the laboratory, the undecomposed and partially decomposed litter of *A. compressus*, consisting of distinguishable fallen leaves, petioles, and branchlets, were separated from the litter of other species, mineral soil, and humus. The litter was shade-dried at room temperature for 10 days. The litter was then ground to powder and packed in polyethylene bags for further use. *A. gangetica* and *P. polystachion* were used as indicator species. These plants were chosen as they represent noxious weed species infesting oil palm plantation. Mature seeds of *A. gangetica* and *P. polystachion* were collected from the same oil palm fields.

### 2.2. Leachate Preparation

Ground *A. compressus *litter (100 g) was steeped in 1 L of distilled water for 18 h at room temperature (25°C) and filtered through a double layer of cheesecloth, followed by filtration through filter paper (no. 1, Whatman). The extract was then diluted with distilled water to obtain concentrations of 10, 20, 30, 40, and 50 g L^−1^. The diluted samples were preserved in a refrigerator at 4°C until used. Distilled water was used as the control, that is, 0 g L^−1^.

**Experiment 1 (bioassay for the litter leachate)**

Healthy-looking weed seeds of uniform size were pretreated with 1.5% (v/v) sodium hypochlorite solution for 1 min for surface sterilization and were then washed three times for 3 min with distilled water. This treatment did not inhibit seed germination. Five milliliters of each litter leachate (10, 30, or 50 g L^−1^) or distilled water (for control) was placed in sterile 9 cm Petri dishes underlaied with two sterile filter papers (no. 2, Whatman) and replicated four times for each concentration. The dishes were kept at room temperature for 2 h to ensure that the temperature of the solution was in equilibrium with the room temperature. Then, twenty-five surface-sterilized seeds of *A. gangetica* or *P. polystachion* were placed on each Petri dish. The Petri dishes were sealed with parafilm to prevent water loss and avoid contamination and incubated at room temperature for 9 days in dark. The germination temperature was about 25°C. The experiment was laid out in a completely randomized design (CRD) with four replications. The number of weed seeds germinating in all the Petri dishes was counted daily up to 9 days. Germination was considered to have occurred when a radicle protruded beyond the seed coat by at least 1 mm. The length of the radicles and hypocotyls was measured after 9 days.

Final germination percent (FGP) and mean germination time (MGT) were calculated using the following formulae:

(1) $$\begin{array}{c} \text{FGP}\left(\%\right)=\frac{\text{Gsd}\times 100}{N}, \end{array}$$

where Gsd is the final number of germinated seeds in the respective treatment and *N* is the number of seeds used in the bioassay, and

(2) $$\begin{array}{c} \text{MGT}\;\left(\text{days}\right)=\frac{\sum Dn}{\sum n}, \end{array}$$

where *n* is the number of seeds germinated on day *D* and *D* is the number of days counted from the beginning of germination.

**Experiment 2 (effect of litter leachate-amended soil on weeds growth)**

The experiment was conducted in 15 cm × 5 cm polybags in a glasshouse at Universiti Putra Malaysia (3° 02′ N, 101° 42′ E; elevation 31 m a.s.l). The experiment was conducted in March 2012. Polybags were kept under glasshouse conditions of 25°C minimum and 32°C maximum, 95 ± 2% relative humidity, and a 12/12 h light/dark regime. The factorial combination of treatments was laid out in a randomized complete block design (RCBD) with four replications. Treatments included six different leachate concentrations of* A. compressus*, namely, 0 (distilled water), 10, 20, 30, 40, and 50 g L^−1^. The test weed species were *A. gangetica* and *P. polystachion*.

Soil for this pot experiment was collected from the oil palm field. Ten *A. compressus*-free spots were selected in the locality, and one soil sample was taken from each spot at a depth of 0–20 cm. The soil was pulverized, and visible pieces of organic matter were removed. Then, a composite soil mass was made by mixing the individual soil masses thoroughly. The soil was then crushed, air-dried, and preserved. Samples of 250 g of soil were placed in each polybag. The soil type was a sandy clay loam (450.2 g/kg clay, 110.3 g/kg silt, and 430.2 g/kg sand and pH = 4.69, CEC = 6.4 cmol kg^−1^, total *N* = 1.2 g/kg, available *P* = 4.1 *μ*g/g, exchangeable *K* = 31 *μ*g/g, exchangeable Ca = 68.3 *μ*g/g, exchangeable Mg = 49.3 *μ*g/g, and organic carbon = 14 g/kg).

The *A. gangetica* and *P. polystachion* seeds were allowed to sprout and germinate for three days at room temperature in dark. The three-day-old weed seedlings were then transplanted into the polybags with ten seedlings per polybag. After transplanting, the litter leachate treatments was applied. Exactly 50 mL of either distilled water (for the control) or *A. compressus* litter leachate (10, 20, 30, 40, or 50 g L^−1^) was added to the soil samples. The root and shoot lengths and seedling dry weights were recorded at the end of two weeks. The* P. polystachion* shoot length (cm) was measured from the ground level to the tip of the longest leaf. The *A. gangetica* shoot length (cm) was measured from the ground level to the tip of the shoot. Shoot and root samples were carefully separated and rinsed in water and then oven-dried at 70°C for 72 h. The total dry matter was calculated by the summation of root and shoot weights.

Leaf total chlorophyll content was estimated using the method of Witham. Fresh leaf from each polybag was cut into pieces using scissors, and 200 mg of cut leaves was transferred into a plastic vial containing 20 mL of 80% acetone. The vial was quickly corked airtight, and kept in the dark for 72 h. Absorbency of the solution was recorded at 645 nm and 663 nm using a scanning spectrophotometer (models UV-3101PC and UV-VIS NIR).

**Experiment 3 (fate of *A. compressus* litter phenolics in the soil)**

The experiment was conducted in 100 cm^3^ plastic containers in a glasshouse (same as Experiment 2) at Universiti Putra Malaysia. Five grams of the *A. compressus* litter was mixed thoroughly with 250 g of soil (same as soil in the Experiment 2), which was placed in 100 cm^3^ plastic containers, and 15 mL of the microbial inoculant was added to each container. The containers were covered with perforated foil and incubated in the dark in the glasshouse. The samples were readjusted gravimetrically to their initial water content at weekly intervals. To facilitate decomposition of the *A. compressus* litter, microbial inoculant was added to each container. The microbial inoculant was prepared by incubating 150 g of fresh soil collected from a cultivated field, with 10 mL of Hoagland's solution, and 15 mL of distilled water in the dark for 4 days. Then, 300 mL of distilled water was added and the supernatant was filtered through Whatman no. 1 filter paper. One set of three pots was removed each time for chemical analysis at 0, 1, 2, 3, 4, and 5 weeks of incubation.

All the soils from each container were extracted by adding 250 mL of distilled water, shaking for 1 h at room temperature, and filtering the extracts through Whatman no. 1 filter paper. The extracts were preserved in a refrigerator at 4°C. The amount of phenolics in the water extract was estimated using the Folin-Ciocalteu assay. For this assay, an aliquot of 1.0 mL of soil extract was placed into a test tube, and 5 mL of 2% Na_2_CO_3_ in 0.1 N NaOH was added and mixed with a test-tube mixer. Five minutes later, 0.5 mL of Folin-Ciocalteu reagent was added, and the solution was mixed again. The absorbance was read using a spectrophotometer (models UV-3101PC and UV-VIS NIR) at 760 nm after 2 h. A standard curve was prepared in a similar manner using a concentration series of gallic acid solutions in water, and then the phenolic concentration in the soil extracts was estimated (as gallic acid equivalent), based on this standard curve. For the estimation of water-soluble phenolics in the plant tissue 5 g of plant tissue was extracted by 50 mL distilled water. For the estimation of acetone extractable phenolics in the plant tissue or soil samples, the same protocol was used (except for the extraction). The extracts were prepared using 70% acetone.

### 2.3. Statistical Analysis

All data were analyzed using the analysis of variance procedure (ANOVA), and means were separated by the Tukey test at the 5% probability level, using Statistical Analysis System software (SAS, version 9.2). Regression analysis was performed to determine the relationship among variables and treatments.

## 3. Results

**Figure 1:**
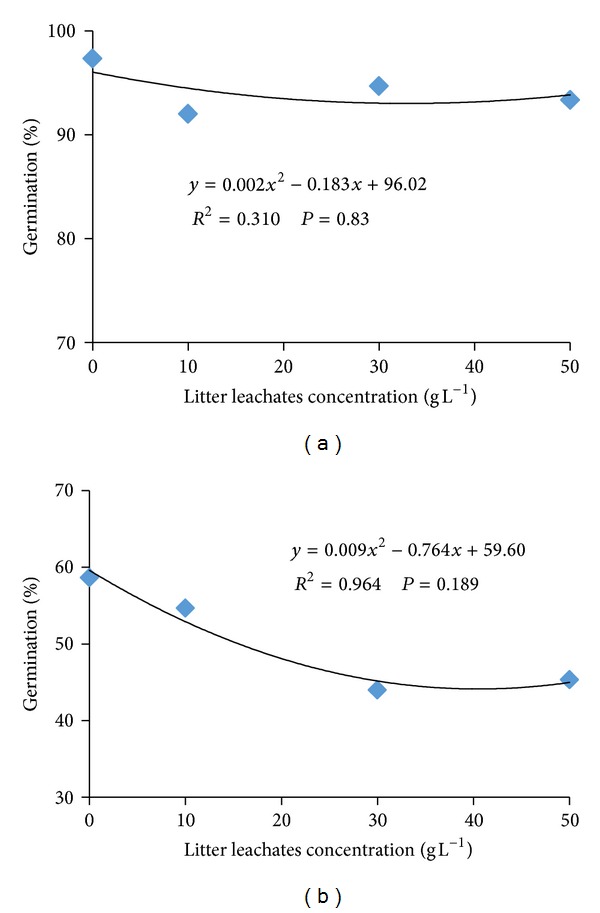
Dose-response relationship curve of the effect of *A. compressus* litter leachates on final germination percent of *A. gangetica *(a) and *P. polystachion* (b).

**Figure 2:**
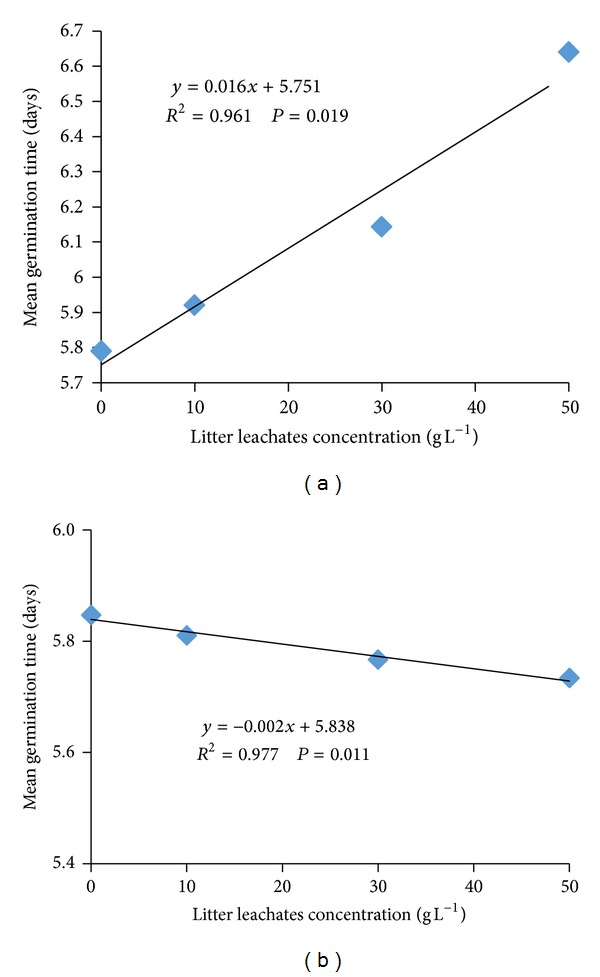
Dose-response relationship curve of the effect of *A. compressus* litter leachates on mean germination time of *A. gangetica* (a) and *P. polystachion* (b).

Final germination percent of *A. gangetica* was not affected by different litter leachate concentrations of the *A. compressus* (Figure 1(a)). The effect of *A. compressus *on *P. polystachion* final germination percent also was not significant (Figure 1(b)). Mean germination time of *A. gangetica *increased significantly and linearly as the *A. compressus *litter concentration increased (Figure 2(a)). The higher value (6.64 days) of *A. gangetica* mean germination time was observed at the highest *A. compressus* leachate concentration of 50 g L^−1^, followed by 30 (6.14 days), 10 (5.9 days), and 0 g L^−1^ (5.7 days) (Figure 2(a)). In *P. polystachion*, the mean germination time decreased significantly linearly with increasing the litter leachate concentrations of the *A. compressus* (Figure 2(b)).

**Figure 3:**
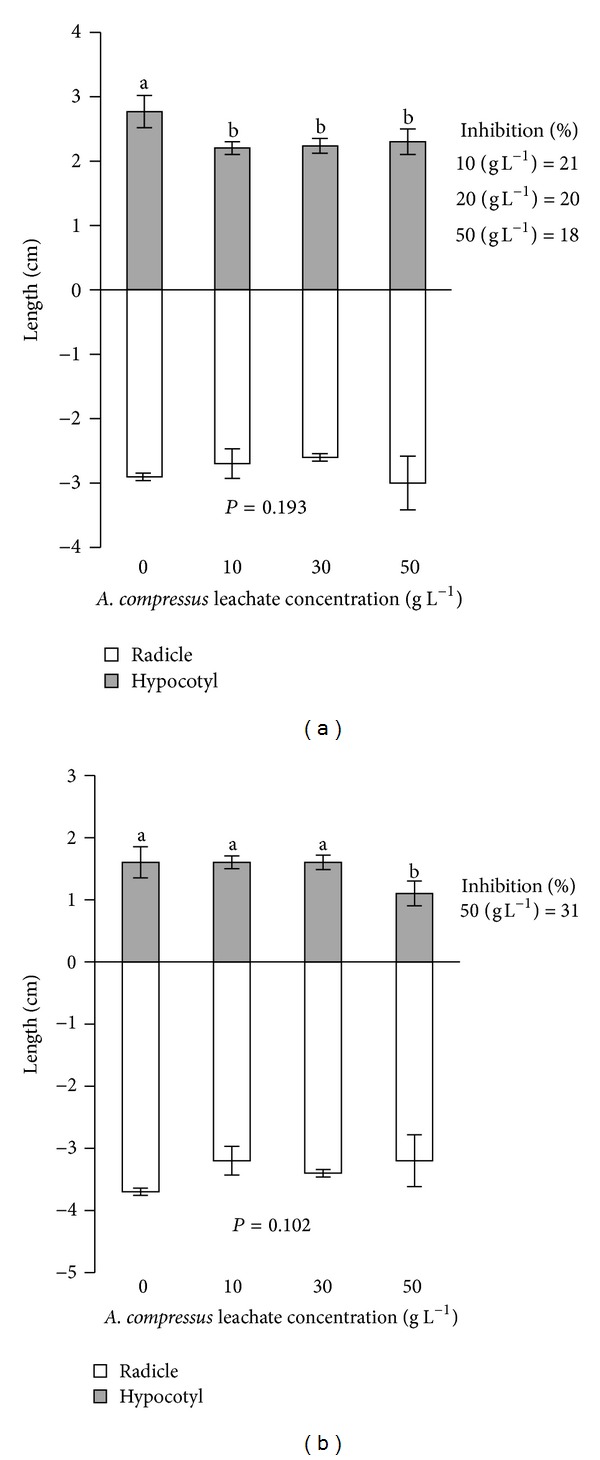
Effect of *A. compressus* litter leachates on seedling growth of *A. gangetica* (a) and *P. polystachion* (b). The error bars are standard deviations (SD) of four replicates. The vertical bars (mean ± SD), denoted with same letters, indicate a nonsignificant difference at *P* = 0.05 according to Tukey's test.

*A. gangetica* hypocotyl length was reduced significantly at all levels of *A. compressus* litter leachates (Figure 3). Hypocotyl length reduction in *A. gangetica* at the 10, 30 and 50 L^−1^ of *A. compressus* compared to the control was 21, 20, and 18%, respectively. *P. polystachion* hypocotyl length only at 50 g L^−1^ level of *A. compressus* litter leachate showed significant differences with the control (0 g L^−1^) (Figure 3). At 50 g L^−1^ level, *P. polystachion* hypocotyl length reduction was 31%. Radical length of *A. gangetica* and *P. polystachion* was not influenced by litter leachates of *A. compressus* (Figure 3).

The *A. compressus* litter leachate-amended soil showed stimulatory effects on the shoot length of *A. gangetica*. The degree of stimulation increased with rising concentrations. However, the *R*-value suggested that the correlation between the leachate concentration and the shoot length was not high (*R* = 0.726, *P* = 0.031). Root length of *A. gangetica* showed a decreasing with increasing *A. compressus* litter leachate concentration in the soil, but this decrease was not significant (*R* = 0.064, *P* = 0.629). Seedling dry weights of *A. gangetica* showed a linearly increasing trend with increasing litter leachate-amended soil concentrations, but the differences were not significant (*R* = 0.022, *P* = 0.780). The *A. compressus* litter leachate-amended soil did not show significant decrease on shoot and root length of *P. polystachion*, respectively (*R* = 0.002, *P* = 0.947 and *R* = 0.0511, *P* = 0.668). Seedling dry weights of *P. polystachion* showed a linearly decreasing trend with increasing litter leachate concentrations, but the correlation between the leachate concentration and the seedling dry weights was not so high (*R* = 0.677, *P* = 0.044). The total chlorophyll content did not vary significantly in the leaves of *A. gangetica* (*P* = 0.667) and *P. polystachion* (*P* = 0.335) due to the different leachate-amended soil concentrations from those of the *A. compressus* treatments.

**Figure 4:**
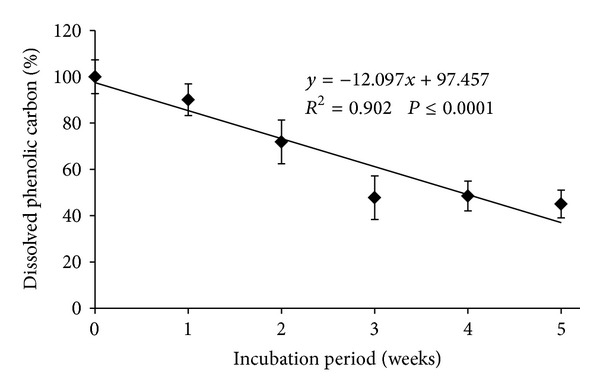
Effect of the decomposition period of *A. compressus* on percent dissolved phenolic carbon. The error bars are standard deviations of the three replicates.

The release pattern and amounts of dissolved phenolic compounds released into the soil by the *A. compressus* litter are presented in Figure 4. The dissolved phenolics in the soil were determined to gain further insight into the changes in the dissolved phenolic compounds released over time. The dissolved phenolics tended to decrease during the 6-week decomposition period (Figure 4). This trend was statistically significant. However, it was clear that during the initial phase (0 week) the concentration of dissolved phenolics was higher and decreased gradually up to 2 weeks, but abruptly decreased at 3 weeks and remained steady until the end of the incubation period. Phenolic content after 1 week was 90.1%, while 71.9, 47.8, 48.5m, and 45.1% percent of dissolved phenolic compounds were obtained at 2, 3, 4, and 5 weeks of incubation, respectively. This implies that phenolic compounds decreased relative to initial time by about 10, 28, 52, 52, and 55% at 1, 2, 3, 4, and 5 weeks after incubation, respectively.

## 4. Discussion

The phytotoxicity of *A. compressus* litter was evaluated using an aqueous extract bioassay on the germination of seeds and growth of young seedlings of weeds. Sites of action for allelochemicals have been reported to include cell division, pollen germination, nutrient uptake, photosynthesis, and specific enzyme function, although seed germination and seedling growth are commonly reduced. The aqueous extracts of *A. compressus* litter did not have either inhibitory or stimulatory effects on the germination of weeds. However, the *A. compressus* litter leachates exhibited a germination delay. This result was similar to that reported by Tesio et al. [17], who found that *Helianthus tuberosus* delayed germination of lettuce without any significant effect on total germination. These findings suggest that reliance on only the total germination value during evaluation of the phytotoxic potential of an allelopathic species against an indicator species may not provide, in some circumstances, an exhaustive picture of the germination dynamics. Even if the results on final germination are similar to those of the control, a different germination pattern may occur. In environments characterized by high competition for resources together with a high weed potential, even small germination delays and the emergence pattern of a species or community may give a competitive advantage over a less-aggressive neighbor and establish a new stability within the plant community after a period of adaptation [18].

Germination of weed seeds was not affected by the aqueous litter leachates of *A. compressus*. However, the hypocotyl lengths of both of the weeds were significantly retarded. Similarly, Casini and Olivero [19] tested the influence of seed leachates, water extracts of residues, and root exudates of legume cover crops, namely, *Pueraria phaseoloides*, *Canavalia ensiformis,* and *Mucuna pruriens* on germination and seedling growth of *Imperata brasiliensis*. The water extracts of shoot residues of all cover crops promoted the germination, while, the germination index was remarkably delayed by 22 and 26% with the highest extract concentration (4%) of *M. pruriens* and *Canavalia ensiformis*, respectively. Also, Oyerinde et al. [20] had observed that the fresh aqueous shoot extracts of *Tithonia diversifolia* did not show allelopathic effects on the germination of *Zea mays*; however, the radicle and plumule lengths of the seedlings were significantly inhibited by the aqueous extract. There was a linear reduction in hypocotyl growth with increasing extract concentration. These results suggest that seeds were able to germinate, but they produced seedlings that were still impaired in growth. The *A. gangetica* growth inhibition started at the lowest applied concentration (10 g L^−1^). The two test species differed in their growth sensitivity to allelopathic interference. The *A. compressus* extracts were more effective at inhibiting hypocotyl growth in *P. polystachion* (grass weed) than in *A. gangetica* (broad-leaved weed). Sahid et al. [21] reported that the germination, radical length, and dry weight of *Asystasia intrusa* (*A. gangetica*) decreased when it grew into full-strength aqueous extract (66.6 g L^−1^) of *Calopogonium caeruleum,* but these characteristics were not affected by the presence of *Centrosema pubescens*.

The extracts inhibited seedling growth of the studied weed species, with a higher inhibition of *P. polystachion* hypocotyl length than that observed for *A. gangetica*. This suggests that attributes of seed germination and seedling growth of the weeds were differentially susceptible to the aqueous extracts of *A. compressus* [22, 23]. The different responses of bioassay species to *A. compressus* litter extracts might be due to evolutionary differences in resistance to allelopathic compounds among the target species [24, 25].

It has been shown that solution phenolic acid concentrations of 100–1000 ppm are allelopathically active and toxic to seedlings [26–28]. In the present study, the concentrations of phenolics in the shoots, litter, and field samples were >100 ppm (Table 1), which suggests that the *A. compressus* investigated had allelopathic potential.

In the present study, the application of *A. compressus* litter leachate-amended soils did not reduce root length and dry weight of two-week-old *A. gangetica* and increased *A. gangetica* shoot length. The shoot and root lengths of *P. polystachion* sown in *A. compressus* litter leachate-amended soils were observed to be similar to those of plants in the control treatment and only decreased seedling dry weight. In addition, the present study revealed that the leaves of litter leachate treated plants looked healthier than the leaves of the control plants, during the first two weeks of the treatment. Oyerinde et al. [20] also reported that *T. diversifolia* may contain allelochemicals that performed both stimulatory and inhibitory functions. *Z. mays *treated with aqueous shoot extracts of *Tithonia diversifolia* accumulated more materials in their development as it was reflected in the shoot height and fresh and dry weights compared to their counterparts in the control regime. A similar growth promoting effect on wheat seedlings was reported by Hussain et al. [29], using Senna mulch as the allelochemical source. Sangakkara et al. [30] also reported that *T. diversifolia* is a potential green manure and organic fertilizer for vegetable crops. This is further corroborated by Ilori et al. [31] who reported the stimulatory effect of *T. diversifolia* on the germination and growth of *Oryza sativa*.

These studies revealed that aqueous *A. compressus* litter extracts could show stimulatory or inhibitory effects on seedling growth of test weeds, depending on planting media (without soil or soil). Similarly, Ohno et al. [27] showed that clover residues significantly decreased radicle growth of wild mustard by 20% at the first sampling after red clover incorporation (8 days after incorporation). Ohno et al. [27] believe that phenolic compounds released by the decomposing clover residues were sorbed and/or oxidized by the soil and hence the sorbed phenolics were found to be much greater than available soluble phenolics. In another study Ohno and Doolan [32] showed that in the absence of soil (sorbents) the phenolic compounds from red clover decomposition were stable throughout the 5-week incubation, in contrast to the shorter period of toxicity in field soils [27]. These results suggest that the sorption process is a key factor in determining the level of phytotoxicity that is observed after residue incorporation. 

A litter incubation study in soil was conducted to gain insight into the fate of allelochemicals from *A. compressus* litter in a soil system after several weeks of incubation under simulated natural conditions. Whitehead et al. [26] concluded that phenolics extracted with water were ecologically more important. Hence, in the present study only the water-soluble fractions of phenolics in the soil were considered.

The results showed that the dissolved phenolics tended to decrease during the 6-week decomposition period. The phenolic content in the soil, which was the product of litter decomposition, was higher at the initial period (0 week), but after two weeks of incubation, the amount of soluble phenolics had declined abruptly and then leveled off over time. The concentration of soluble phenolics in the soil was 90 and 72% after the first and second week of incubation and 55% after the sixth week. Other authors have also reported the fast disappearance of phenolics within the first week or month. Shofield et al. [33] reported the disappearance of more than 50% of phenolics from willow leaves within 2 weeks of incubation. Rashid et al. [34] reported 69% soluble phenolics in the soil after the first week of incubation of kudzu litter and a 62% phenolic content after the sixth week. The reduction in phenolics content over the five-week period suggests that microbes were utilizing the phenolic compounds during the incubation period [32]. 

After the death of plants, phenolics may persist for weeks or months and effect decomposer organisms and decomposition processes in soils [35]. Ohno et al. [27] reported that red clover phenolics remained in the soil solution for up to several weeks. However, the phytotoxicity was present only in the immediate sampling after red clover incorporation (8 days after incorporation). This is probably because of the close relationship between phytotoxicity of phenolic acids [36] and oxidation reactivity with soils [37].

From an allelopathic perspective, phytotoxic compounds are not considered suitable if they are not released into their environment, and the fate of the allelochemical in the soil should be considered. Once an allelochemical or a mixture of allelochemicals enters a soil system, processes such as adsorption-desorption, microbial decomposition, and leaching can modify its behavior [38]. The phytotoxic activity of allelochemicals in soil is, therefore, a function of complex interactions among soil and plant factors. 

The results of the bioassay, growth, and litter incubation studies support the conclusion that *A. compressus* litter might interfere allelopathically with its neighboring species. However, the allelopathic effects depend on weed species and soil characteristics. The allelopathic action also may not persist for a considerable period of time.

## Figures and Tables

**Table 1:** Water and acetone extractable phenolics in *A. compressus* tissues and soil at different sampling dates.

Treatment	Phenolic compounds in soil (ppm.)			
	12MAP		24MAP	
	Water extractable	Acetone extractable	Water extractable	Acetone extractable
*A. compressus* soil	2.9*	71.6	3.4	353.0
*A. compressus* litter	—	—	150	180
*A. compressus* shoot	—	—	400	423

MAP: Months after planting *A. compressus* in the field, *Data are average of 3 replicates.
